# A paradigm shift in the quantification of wave energy attenuation due to saltmarshes based on their standing biomass

**DOI:** 10.1038/s41598-022-18143-6

**Published:** 2022-08-16

**Authors:** Maria Maza, Javier L. Lara, Iñigo J. Losada

**Affiliations:** Instituto de Hidráulica Ambiental de la Universidad de Cantabria (IHCantabria), Isabel Torres 15, 39011 Santander, Spain

**Keywords:** Civil engineering, Ecosystem services, Ocean sciences, Fluid dynamics

## Abstract

Most existing analytical and numerical models to quantify wave energy attenuation attributed to saltmarshes are based on the definition of a drag coefficient that varies with vegetation and wave characteristics and requires calibration, i.e., a case-specific variable. With the aim of determining a new variable to estimate wave energy attenuation without the use of calibration coefficients, wave attenuation caused by different saltmarsh species and the relationship with the ecosystem standing biomass are experimentally studied. Samples of four real saltmarshes with contrasting morphological and biomechanical properties, namely, *Spartina* sp., *Salicornia* sp., *Halimione* sp. and *Juncus* sp., are collected in the field and placed in a wave flume for testing under different regular and random wave conditions. Two meadow densities are considered, in addition to zero-density cases. Thus, wave damping coefficients are obtained in vegetated cases, *β*, and bare soil cases, *β*_*B*_, and wave damping produced solely by the meadow standing biomass, *β*_*SB*_, is determined. The obtained wave damping coefficients are related to a new variable, the hydraulic standing biomass (*HSB*), which is defined as a function of the meadow mean height and standing biomass and incident flow characteristics. Linear fitting relationships between the wave damping coefficient and *HSB* are obtained, allowing *β* and *β*_*SB*_ estimation without the need for calibration. Therefore, the use of these new relationships facilitates direct quantification of wave energy attenuation due to saltmarshes based on incident wave conditions, mean plant height and meadow standing biomass, variables that can be obtained from aerial images or remote sensing data, extending the applicability of the approach. Another key aspect is that this approach does not depend on any calibration coefficient and can be directly applied with knowledge of the abovementioned characteristics. This may represent a paradigm shift in the estimation of wave energy attenuation attributed to saltmarshes.

## Introduction

Coastal communities worldwide face an increasing risk of flooding as a result of the rising sea level, increasing storm intensity and land subsidence^1,2^. In temperate zones, stable intertidal mudflats support stands of saltmarshes. These saltmarshes function as natural buffer zones, providing protection from waves^3–7^. Their associated coastal protection service benefits millions of people, yielding significant socioeconomic gains^8–11^. It should also be noted that flood protection through saltmarsh conservation and restoration can provide a more sustainable, cost-effective and environmentally friendly alternative to conventional coastal engineering^5,12,13^ and that the use of saltmarsh fronting levees can reduce global coastal protection costs^14^. A very important aspect in quantifying the wave energy dissipation capacity of these ecosystems is the correct definition of ecosystem characteristics^15,16^. Thus, it is crucial to adequately consider these characteristics to properly quantify their coastal protection service.

The biotic structures in these ecosystems determine their protection capacity, since these structures influence the momentum loss, resulting in wave energy attenuation^17^. Thus, existing analytical and numerical models for the quantification of wave energy attenuation due to vegetation define vegetation through these structures. In particular, the stem diameter and number of stems per unit area are the most commonly used vegetated ecosystem features in analytical^18,19^ and numerical models^20–23^ used to quantify the wave energy damping capacity. Additionally, these models rely on the definition of a drag coefficient, which is commonly used as a calibration coefficient considering all aspects, such as the plant morphology complexity or flexibility, not directly introduced in the modeling process. Moreover, this drag coefficient varies with both wave and vegetation characteristics, requiring calibration and yielding a case-specific variable^15^. To overcome this limitation and obtain a prediction equation that does not require calibration, Maza et al.^24^ proposed a new model based on the ecosystem submerged solid volume fraction that was later applied to quantify the wave attenuation effect of Rhizophora mangrove forests^25^. However, this model has only been applied to rigid elements but does not consider the stem flexibility, a relevant aspect in most vegetated ecosystems determining the attenuation capacity^26,27^. Looking for a better characterization of wave interaction with flexible vegetation, recent work has been developed based on the definition of the Cauchy number and a buoyancy term defined as a function of plant density, plant volume, plant elastic modulus and the second moment of inertia of the stem. These contributions are based on the definition of an effective stem length considering a drag coefficient as that of a rigid stem under a stationary unidirectional flow (e.g.^28,29^) or resolve the plant motion as a function of drag and restoring forces that are defined based on the aforementioned vegetation parameters (e.g.^30,31^). Although they represent a major breakthrough in parameterizing the physics involved in the interaction of waves with flexible vegetation, they require the correct definition of all the aforementioned parameters, such as plant density, flexibility and detailed geometry, which requires a great effort. Besides, they have been obtained from experiments with vegetation mimics representing a single plant species. Therefore, complete characterization of a given vegetated ecosystem includes measurement of leaf traits, biomechanical properties of plants and number of individuals per unit area. Additionally, these properties vary seasonally for most saltmarsh species, leading to a highly notable change in the corresponding coastal protection capacity^32,33^ and involving much measurement effort. Therefore, a new approach to estimate ecosystem properties that involves less effort while avoiding calibration coefficients, such as the drag coefficient, is needed to adequately quantify the ecosystem wave attenuation capacity under different scenarios.

An interesting finding was presented by^34^ for two species with contrasting biomechanical properties (*Puccinellia maritima* and *Spartina anglica*) where they conclude that plotting wave attenuation as a function of standing biomass, defined as the above-ground dry weight of vegetation per unit area, shows that wave attenuation is remarkably similar for flexible and rigid vegetation when considered on a biomass basis. This aspect was also presented by^35^ for the same two species and different meadows densities. They showed a positive relationship between wave height attenuation and standing biomass for the two species with contrasting Young's modulus. This finding established that the standing biomass can represent a unique variable defining the ecosystem wave energy attenuation capacity, overcoming the need for calibration coefficients and intensive efforts in plant trait characterization. Additionally, the standing biomass is a variable that has been widely studied by ecologists and biologists^36–38^ and can be estimated via remote sensing techniques or aerial images^39–41^, which highly facilitates its estimation in different saltmarshes at different times. Therefore, this study aimed to further explore this promising finding that could allow quantification of the wave attenuation effect of different species of saltmarshes during different seasons via the use of the corresponding standing biomass. To this end, a set of experiments using real vegetation with contrasting morphological and biomechanical properties subject to different incident wave conditions was performed. The experimental campaign resulted in a new formulation allowing estimation of wave attenuation as a function of the meadow standing biomass. This relationship could help to quantify the expected coastal protection provided by different saltmarshes species without the use of calibration coefficients, incentivizing saltmarsh conservation and restoration.

## Results

### Experimental set-up

**Figure 1:**
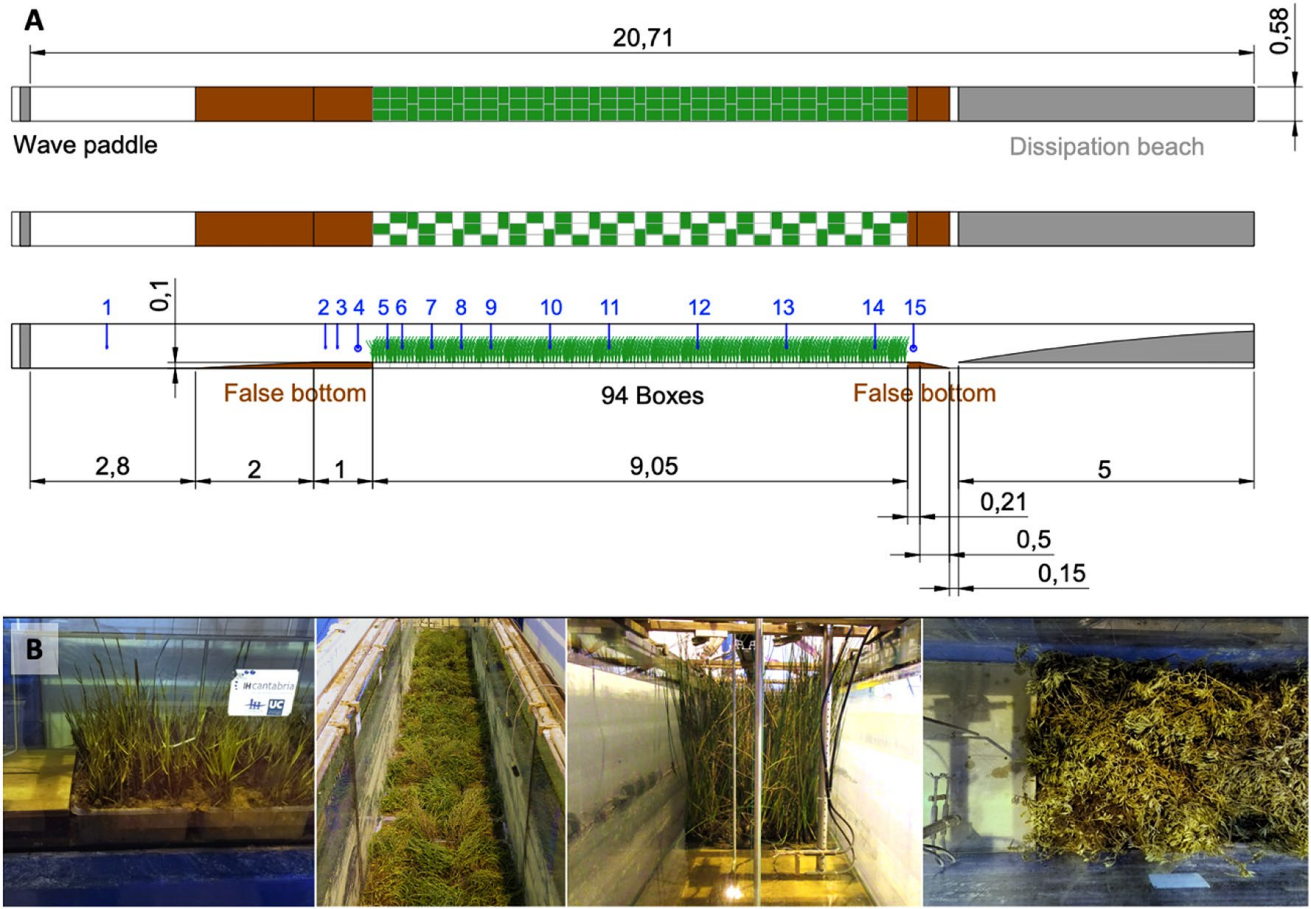
(**A**) Shows a sketch of the experimental flume, where the vegetation box distribution in the 100% and 50% density cases is displayed in the two upper panels and a lateral view in the bottom panel. The green boxes indicate the vegetated area in each case. Free surface sensors are displayed by blue lines and numbers. (**B**) Shows the four species within the flume. From left to right: view of the *Spartina* sp. frontal edge, aerial view of *Salicornia* sp., frontal view of *Juncus* sp. and top view of the *Halimione* sp. rear edge.

Four vegetation species were selected: *Spartina maritima*, *Salicornia europaea*, *Halimione portulacoides* and *Juncus maritimus*. These species were chosen for a broad representation of the biomechanical properties and morphological characteristics of saltmarsh species^42,43^. Plants were collected in Cantabrian estuaries in late summer and early autumn (from early September to late October) during low tide (please refer to the “Methods” section). A total of 105 boxes were collected, of which 94 boxes were used to build a 9.05 m long and 0.58 m wide meadow in a flume (Fig. 1). Five boxes were used to directly estimate the meadow standing biomass in the field (Sample 1 in Table 1), leaving 6 extra boxes for possible contingencies.

**Table 1:** Standing biomass (g/m^2^) and plant height (m) for the four species.

Standing biomass	*Spartina* sp. (g/m^2^)	*Salicornia* sp. (g/m^2^)	*Juncus* sp. (g/m^2^)	*Halimione* sp. (g/m^2^)
Sample 1	310.3	689.7	1674.9	1394.9
Sample 2	279.6	822.6	1974.6	1599.0
Sample 3	304.9	576.7	1767.7	1404.5
Sample 4	308.3	604.0	1811.3	1292.7
Sample 5	348.2	755.6	2061.7	1283.6
Mean (SD)	310.3 (24.7)	689.7 (102.6)	1858.0 (157.3)	1394.9 (127.1)

Plant height	*Spartina* sp. (m)	*Salicornia* sp. (m)	*Juncus* sp. (m)	*Halimione* sp. (m)
Mean (SD)	0.170 (0.074)	0.175 (0.070)	0.714 (0.127)	0.187 (0.113)

Sample 1: 5 boxes from the field; Sample 2: 5 boxes at the leading edge of the meadow in the first cut; Sample 3: 5 boxes at the central part of the meadow in the first cut; Sample 4: 5 boxes at the leading edge of the meadow in the second cut; Sample 5: 5 boxes at the central part of the meadow in the second cut. The mean value and standard deviation (SD) of the standing biomass and plant height are displayed.

Experiments were conducted in a flume 20.71 m long and 0.58 m wide at the University of Cantabria. The flume is equipped with a piston wave maker at its left end and a dissipation beach at the rear end. The 94 vegetation boxes used to create a meadow were introduced into the flume following the pattern shown in panel A of Fig. 1 to minimize any edge effects along the edges of the boxes. To ensure a smooth transition from the bottom of the channel to the vegetated area, two false bottoms were constructed with wood, and a thin sediment layer was glued to the wood to mimic the field roughness.

Three meadow densities per species were considered. The meadow density directly determined in the field was chosen under the 100% density scenario. To consider a second meadow density, and therefore a second standing biomass value, plants were removed from half of the boxes following the pattern shown in Panel A of Fig. 1 to prevent creating preferential flow channels along the meadow. This case was considered the 50% density scenario. The study of these two biomass scenarios for each vegetation species is carried out with the aim of covering a wide range of standing biomass values, including low values that may be more representative of meadow winter conditions, thus facilitating the applicability of obtained results. Finally, a second cut was made, in which all plants were removed, resulting in the final scenario with a zero density. Plants were cut from above to avoid any damage along the meadow surface (as shown in Supplementary Fig. S2). In each cut, plants in 5 boxes along the leading edge and in 5 boxes at the center of the meadow were collected to quantify the standing biomass (Samples 2 and 3 for the first cut and Sample 4 and 5 for the second cut in Table 1). Therefore, the standing biomass could be monitored throughout the entire duration of the experiments, from the field until the second cut, when all plants were removed.

Once located in the flume, the meadow was evaluated under regular and random wave conditions considering three water depths, i.e., h = 0.20, 0.30 and 0.40 m. Regular waves were generated using Stokes II-, III- and V-order and Cnoidal theories when applicable. Wave heights ranging from 0.05 to 0.15 m and wave periods varying between 1.5 and 4 s were considered. Random waves were generated using a Jonswap spectrum with a peak enhancement factor of 3.3, a significant wave height varying between 0.05 and 0.15 m and a peak wave period ranging from 1.8 to 4.8 s (please refer to Supplementary Table S1). Additionally, all wave conditions were considered under the zero-density scenario with bare soil for each species. The wave height evolution along the flume was recorded using 15 capacitive free surface gauges, as shown in Fig. 1 (please refer to Supplementary Table S2 for detailed coordinates).

### Meadow characteristics analysis

The characteristics of the vegetation meadows were analyzed by measuring the standing biomass throughout the full duration of the experiments and by measuring the individual plant height (please refer to the “Methods” section). The mean standing biomass value obtained for each species was considered the value associated with the 100% density scenario. Then, half of the standing biomass value was considered under the 50% density scenarios since half of the boxes was randomly cut, and the standing biomass values obtained after the second cut agreed with those obtained after the first cut and in the field, as indicated in Table 1. The plant height for each species was also measured (please refer to the “Methods” section), and the resultant mean value detailed in Table 1 was considered.

### Wave height attenuation analysis

**Figure 2:**
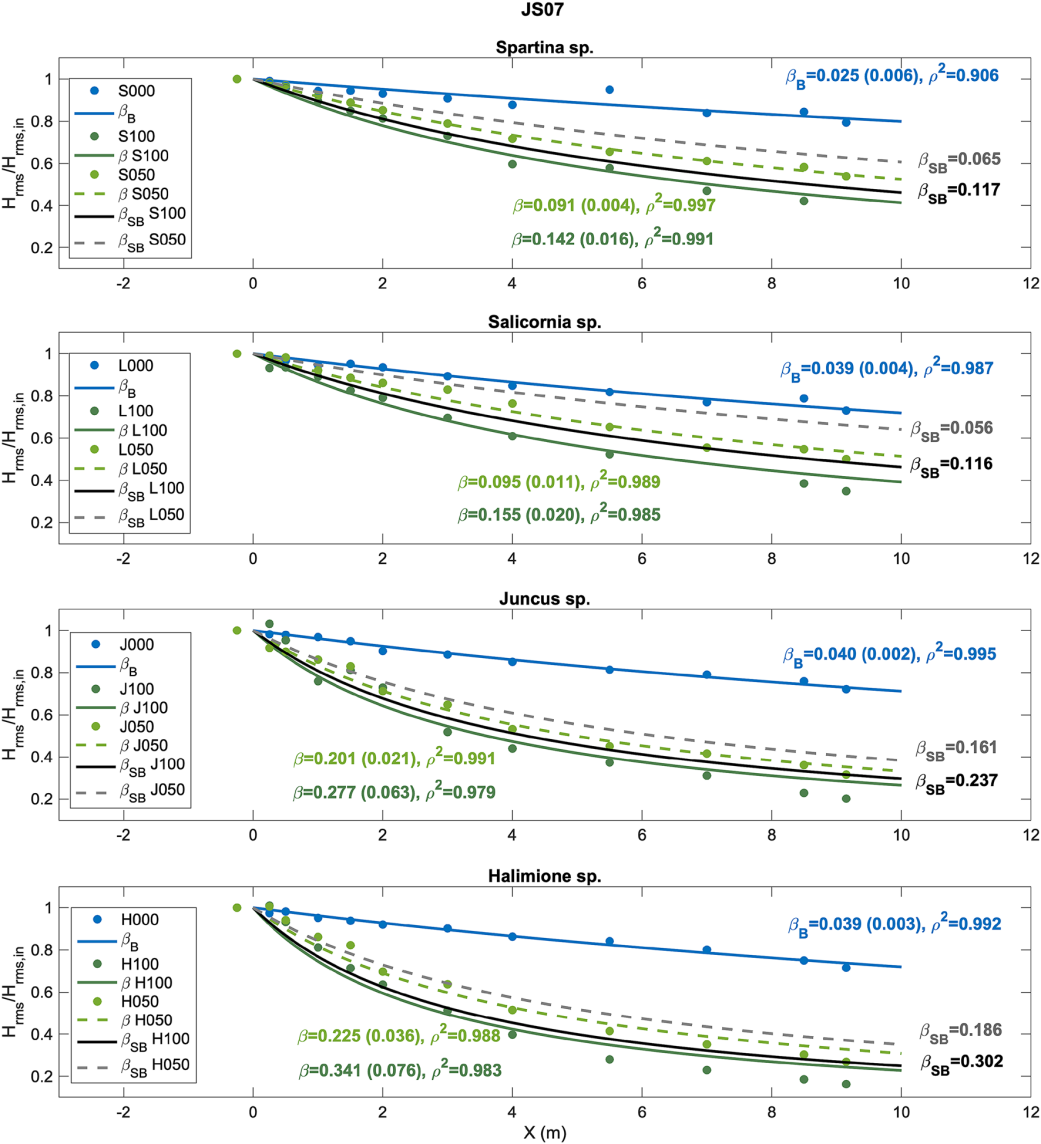
Analysis of wave attenuation under wave condition JS07 for *Spartina* sp. 100% (S100), 50% (S050) and zero density (S000); *Salicornia* sp. 100% (L100), 50% (L050) and zero density (L000); *Juncus* sp. 100% (J100), 50% (J050) and zero density (J000); and *Halimione* sp. 100% (H100), 50% (H050) and zero density (H000). The damping coefficients for the bare soil cases, ${\beta }_{B}$, are displayed in blue. The damping coefficients for the 100% and 50% density cases, $\beta$, are displayed in dark and light green, respectively. The damping coefficients obtained after subtracting the dissipation obtained in the bare soil cases, ${\beta }_{SB}$, are displayed in black and dark gray. 95% confidence interval is shown in brackets and correlation coefficient (${\rho }^{2}$) for each fit is also displayed.

Wave height attenuation analysis was performed following previous studies reported in the literature assessing the capacity by fitting a damping coefficient^6,7,35,44^. The^18^ formulation was used for regular waves, and that of^19^ was used for random waves (please refer to the “Methods” section). Cases with a zero density were also considered in this analysis to quantify the influence of bare soil friction by determining the corresponding damping coefficient, ${\beta }_{B}$. Consequently, β was obtained in the 100% and 50% density cases and the cases without vegetation (please refer to Supplementary Tables S3, S4 and S5 to find the obtained coefficients for all cases). This allowed the determination of a new damping coefficient isolating the effect of the standing biomass, ${\beta }_{SB}$, following^24^ (please refer to the “Methods” section). Figure 2 shows an example of wave height attenuation analysis for the four species and the different densities under wave condition JS07 (Supplementary Table S1).

The damping coefficients for the bare soil cases shown in Fig. 2, ${\beta }_{B}$, are consistent with the soil properties observed in the field. *Spartina* sp. was collected in a muddy area, whereas the other three species were collected in areas with coarser sediments and exhibited a mixture of sand and mud. For all species, wave dissipation was significantly higher under the 100% density scenario than that under the 50% density cases, as expected, highlighting the importance of the standing biomass in wave energy dissipation. It was also observed that bottom friction-induced dissipation plays a more important role for the pioneer species, i.e., *Spartina* sp. and *Salicornia* sp., than for the upper marsh species, i.e., *Juncus* sp. and *Halimione* sp., which can dissipate wave energy to a greater extent.

**Figure 3:**
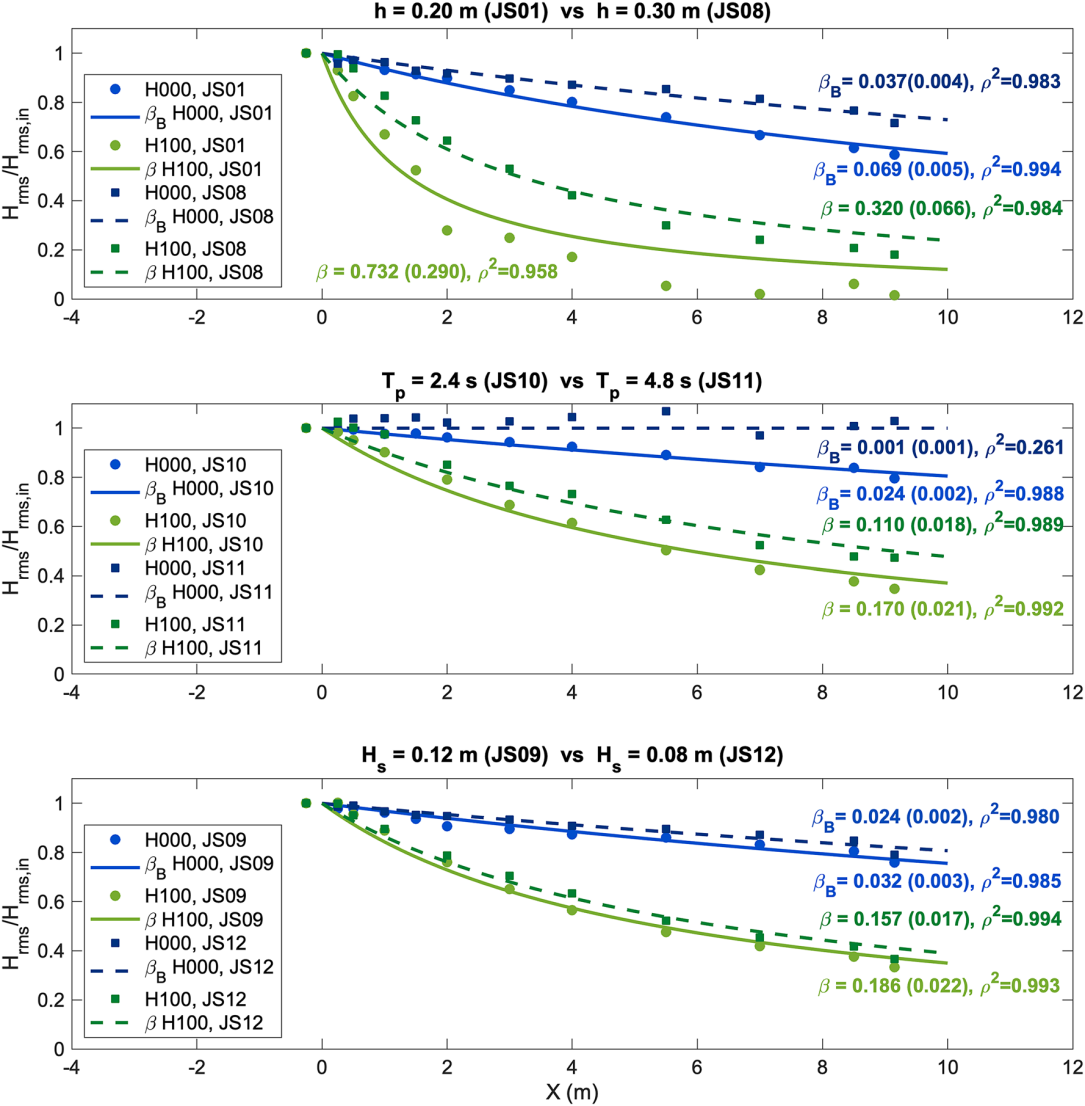
Analysis of wave attenuation under the different irregular wave conditions for the *Halimione* sp. 100% (H100) and zero-density (H000) cases. The top panel shows two cases with different h but equal H_s_ and T_p_ values (JS01 and JS08), the middle panel shows two cases with different T_p_ but equal h and H_s_ values (JS10 and JS11), and the bottom panel shows two cases with different H_s_ but equal h and T_p_ values (JS09 and JS12). 95% confidence interval is shown in brackets and correlation coefficient (${\rho }^{2}$) for each fit is also displayed.

The importance of wave parameters in the resultant wave attenuation has been highlighted by several works in the literature. Therefore, not only vegetation characteristics but also incident wave conditions determine the coastal protection capacity. Figure 3 shows a comparison of the obtained wave height attenuation due to *Halimione* sp. under the different wave conditions*.*

The top panel in Fig. 3 shows two cases where H_s_ and T_p_ are equal, i.e., JS01 and JS08 in Supplementary Table S1, and two water depths are considered, namely, h = 0.2 and 0.3 m. As can be observed, wave damping is higher for the smallest water depth, where most of the water column is covered by vegetation since the mean vegetation height for *Halimione* sp. reaches 0.187 m (Table 1). The importance of the water depth with respect to the plant height in terms of wave height attenuation has been reported by several authors^44–46^ who have highlighted this aspect based on the submergence ratio, i.e., the plant height divided by the water depth, revealing higher attenuation at lower submergence ratios on a consistent basis. Bottom friction attenuation is also higher for the smallest water depth, as expected.

The middle panel of Fig. 3 shows two cases with equal h and H_s_ but different T_p_ values, namely, JS10 and JS11 in Supplementary Table S1. Wave height attenuation is higher for the shortest wave period, as well as the damping produced by bottom friction. This is in line with previous studies, such as^35^ and^44^, who conducted experiments involving simulated and real saltmarshes, respectively. Finally, the bottom panel of Fig. 3 shows two cases with different H_s_ but equal h and T_p_ values, i.e., JS09 and JS12 in Supplementary Table S1. As widely reported in the literature, e.g.,^7,47,48^, wave height attenuation increases with the wave height, as shown in the bottom panel of Fig. 3. Bottom friction also increases with the wave height, as expected.

A set of damping coefficients was obtained via the 288 tests conducted in the laboratory, 144 tests involving regular waves and 144 tests involving random waves. Additionally, in all cases, the damping coefficient considering the isolated effect of the standing biomass, ${\beta }_{SB}$, was determined. The relationship of these damping coefficients to the measured standing biomass is explored in the next section with the aim of establishing a new relationship to estimate the wave damping effect of the different saltmarsh species based on the standing biomass, without the need for data fitting.

### Wave damping coefficient as a function of the standing biomass

The mean standing biomass obtained for the different species, Table 1, is considered here to analyze the relationship with the wave damping coefficients obtained by fitting^18^ formulation to wave heights measured along the meadow for regular waves and^19^ formulation for random waves. The plant height was highly variable among the different species (Table 1), ranging from 0.170 m for *Spartina* sp. to 0.714 m for *Juncus* sp. Then, some species were submerged at all tested water depths, while other species remained above water in all tests. In the latter cases, there remained a portion of each plant above the water level, thus not contributing to wave attenuation. To consider the actual interaction between the standing biomass and flow conditions and assuming a uniform vertical distribution, the effective standing biomass, $ESB$, can be defined as follows:

1 $$ESB=DryWeight*\frac{min\left\{{h}_{v},h\right\}}{{h}_{v}}$$

where $DryWeight$ denotes the measured dry weight for each species (g/m^2^), ${h}_{v}$ is the mean plant height and $h$ is the water depth. Additionally, in the submerged cases, the same $ESB$ value will impact flow differently depending on the submergence ratio, $SR$, as defined in Eq. (2). To consider this effect, the standing biomass ratio, $SBR$ in Eq. (3), can be defined as follows:

2 $$SR=\frac{{h}_{v}}{h}, \;\;where \;\; SR=1 \;\;for \;\;{h}_{v}>h$$

3 $$SBR=ESB*SR$$

**Figure 4:**
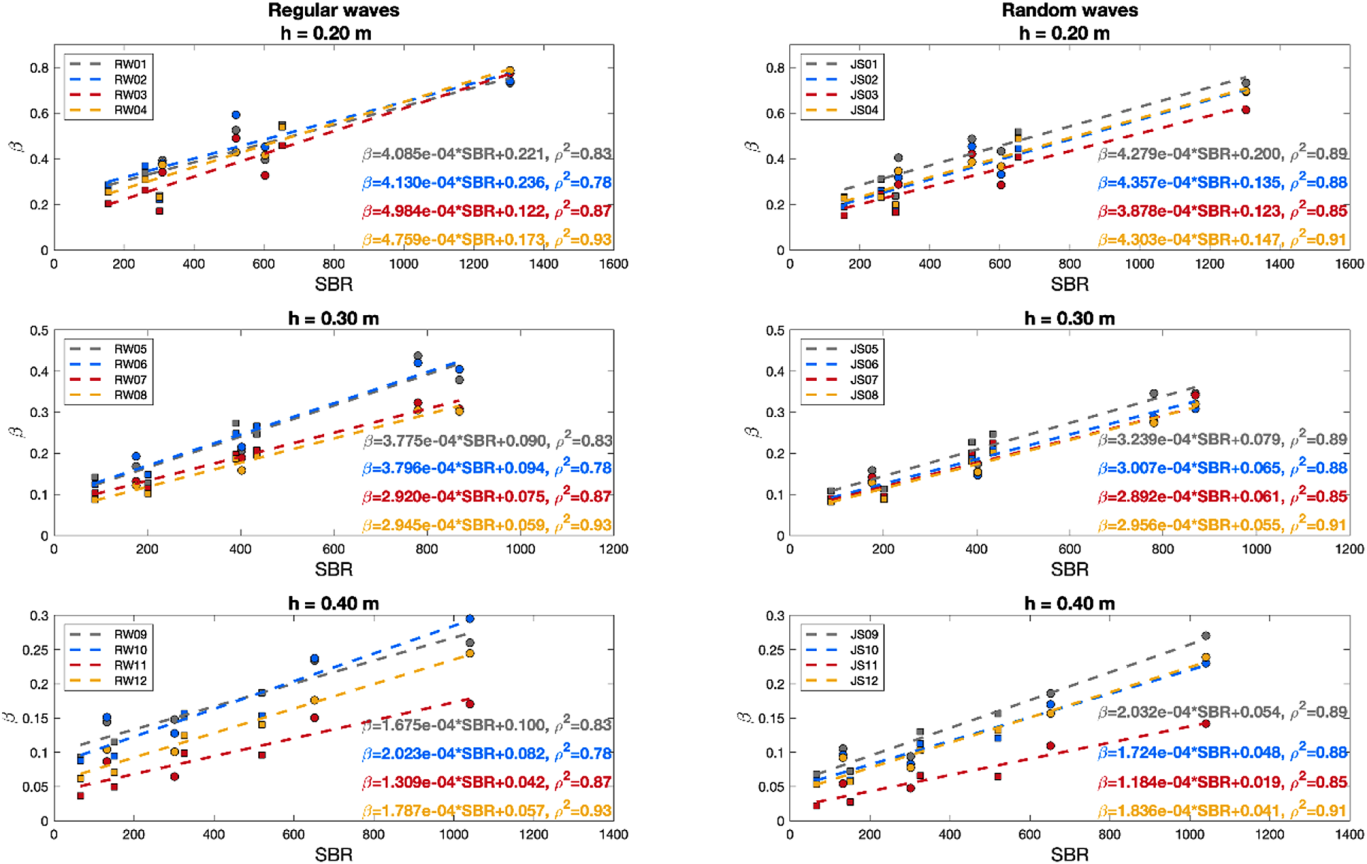
Wave damping coefficient, $\beta$, as a function of the standing biomass ratio, $SBR$, under all regular (left panels) and random (right panels) wave conditions. Each panel shows the wave trains assessed at each water depth, h = 0.20, 0.30 and 0.40 m. The results for the 100% density case are marked with circles and those for the 50% density case are marked with squares. The linear fitting results obtained under each wave condition are also displayed.

Figure 4 shows the relationship between $SBR$ and the measured wave damping coefficient, $\beta$. The results for regular and random waves are displayed for each water depth, and a linear fit was found under each condition.

Under each wave condition, a linear fitting relationship between $\beta$ and $SBR$ was obtained for the eight $SBR$ values, as shown in Fig. 4. For similar $SBR$ values, the highest $\beta$ values were consistently obtained at the smallest water depth, highlighting the notable influence of this parameter on the obtained wave attenuation. Following previous works, such as those of^24^ and^25^, who considered the vegetation submerged solid volume fraction to estimate the resulting wave attenuation and established a common relationship for different water depths, the volumetric standing biomass, $VSB$, can be defined as follows:

4 $$VSB= SBR*\frac{1}{h}$$

**Figure 5:**
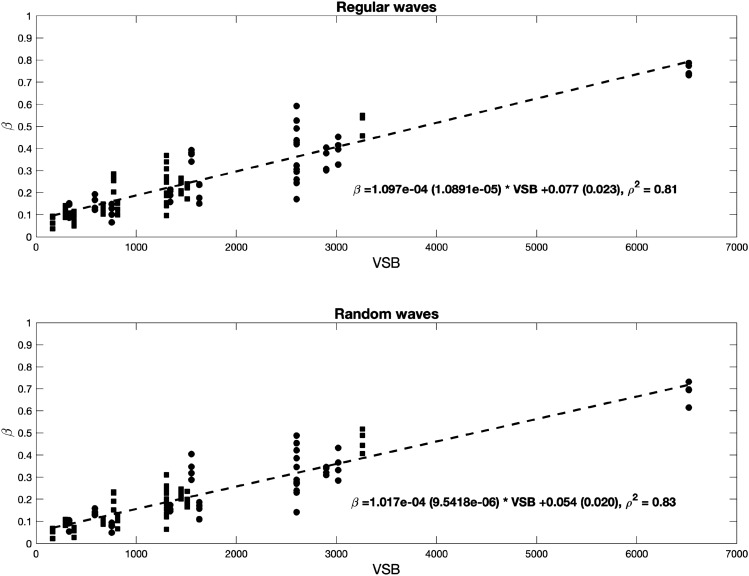
Wave damping coefficient, $\beta$, as a function of the volumetric standing biomass, $VSB$, under all regular (top panel) and random (bottom panel) wave conditions. The obtained linear fitting results are displayed in both panels. 95% confidence interval is shown in brackets and correlation coefficient (${\rho }^{2}$) for each fit is also displayed.

$VSB$ is expressed in units of g/m^3^, which is the weight per unit volume. Exploring the relationship of $\beta$ with this new parameter, it was found that the results for the three water depths could be fitted with a single linear relationship, as shown in Fig. 5. However, despite the linear trend observed in Fig. 5, notable data scatter was observed for each $VSB$ value. Each of these groups corresponds to a certain water depth and $SBR$ value, which were determined under different wave heights and wave periods.

Finally, to account for the characteristics of the incident wave conditions, including the wave height and period, two nondimensional parameters were considered. The first parameter, considering the wave height, is the relative wave height, defined as the ratio of the incident wave height to the water depth, $H/h$. Previous studies have highlighted the importance of this parameter in the resultant wave attenuation (e.g.^24,44^). Under random wave conditions, the considered wave height is ${H}_{rms}$, according to wave attenuation analysis. The second parameter, considering the effect of the different wave periods and the importance of the number of wave lengths inside the vegetation length^49^, is the relative meadow length, defined as the ratio of the meadow length to the wave length, ${L}_{v}/L$. To ensure consistency with the above wave attenuation analysis, in which the wave damping amount per unit length was obtained, the unit meadow length was considered here. Thus, the hydraulic standing biomass, $HSB$, can be defined as:

5 $$HSB=VSB*\frac{H}{h}*\frac{{L}_{v}}{L}$$

**Figure 6:**
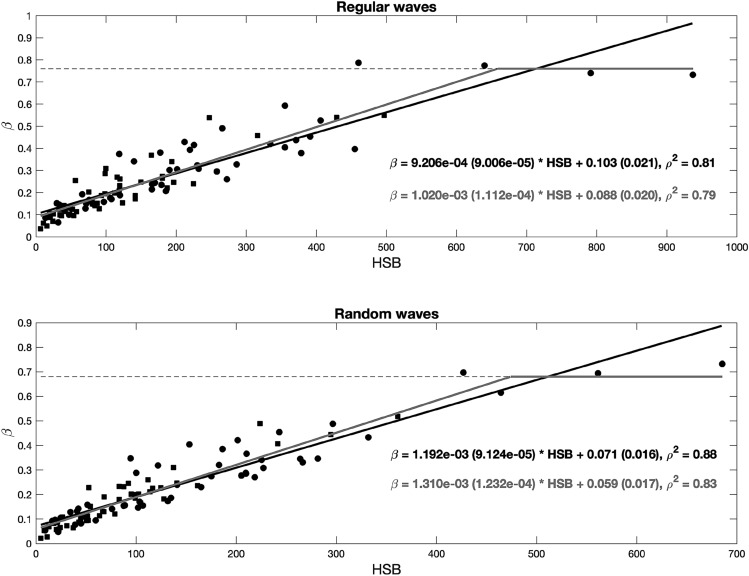
Wave damping coefficient, $\beta$, as a function of the hydraulic standing biomass, $HSB$, under all regular (top panel) and random (bottom panel) wave conditions. Both panels show linear fitting results obtained without considering the saturation point, indicated by the black solid line, and those obtained considering the saturation point, indicated by the gray solid line. The black dashed line indicates the saturation point. 95% confidence interval is shown in brackets and correlation coefficient (${\rho }^{2}$) for each fit is also displayed.

Figure 6 shows the relationship obtained between $\beta$ and this new variable under all regular and random conditions following the linear fitting relationship of $\beta =A*HSB+B$, where $A$ and $B$ are fitting constants with units of (g/m^2^)^−1^ and m^−1^, respectively.

The linear fitting results obtained between $\beta$ and $HSB$ under regular and random wave conditions are shown in Fig. 6 as solid black lines and expressed as Eqs. (6) and (7), respectively, where values between brackets are the 95% confidence interval for each coefficient.

6 $$\beta =9.206\cdot {10}^{-4} \left(9.006\cdot {10}^{-5}\right)*HSB+0.103 (0.021)$$

7 $$\beta =1.192 \cdot {10}^{-3} \left(9.124 \cdot {10}^{-5}\right)*HSB+0.071 (0.016)$$

The inclusion of incident wave condition characteristics reduces the resulting data scatter, highlighting the role of the wave height and period in the obtained wave attenuation, as described in the previous section. An interesting aspect observed in Fig. 6 is that the four cases with the highest wave damping coefficients yielded similar values for the different $HSB$ values. Under regular wave conditions, the mean $\beta$ value for these four cases is 0.76, and under random wave conditions, the value reaches 0.68. This may indicate that the damping coefficient has reached its maximum value and no longer increases with increasing $HSB$ value. To analyze this aspect in more detail, the wave height evolution measured for the four tests in which $\beta$ reaches its maximum value are plotted (as shown in Supplementary Fig. S3). These tests correspond to *Halimione* sp. with a density of 100% and the shallowest water depth, h = 0.20 m. This species achieved the highest standing biomass value among the species considered in these experiments, and for h = 0.20 m, almost the entire water column was covered by vegetation. For these tests, a notable wave height attenuation was observed, where the wave height strongly decayed along the first 5 m of vegetation, and the wave height entirely dissipated along the last 4 m (as shown in Supplementary Fig. S3). The wave damping equation cannot suitably reproduce the strong wave decay within this few meters. Then, an almost constant wave damping coefficient value is reached under the different considered wave conditions, and a saturation regime is observed, in which the wave height beyond the meadow can be assumed to be negligible. To consider this phenomenon, a two-section fitting relationship is proposed, as shown in Fig. 6. The value of the saturation damping coefficient, chosen as the mean value of the four cases analyzed, is plotted as a dashed gray line, and a linear fit is obtained for the remaining data. The two-section fitting relationship is expressed in Eqs. (8) and (9) for both regular and random waves, respectively, where values between brackets are the 95% confidence interval for each coefficient.

8 $$\beta =\left\{\begin{array}{ll}1.020 \cdot {10}^{-3}\left(1.112 \cdot {10}^{-4}\right)*HSB+0.088 \; (0.020) \\ 0.758\; (0.027)\end{array}\right. \begin{array}{l} \;\;0 < HSB < 659\\ \;\; HSB > 659\end{array}$$

9 $$\beta =\left\{\begin{array}{l}1.310\cdot {10}^{-3}\left(1.232\cdot {10}^{-4}\right)*HSB+0.059\; (0.017) \\ 0.684 \;(0.066)\end{array}\right. \begin{array}{l}\;\;0<HSB< 474\\ \;\; HSB>474\end{array}$$

**Figure 7:**
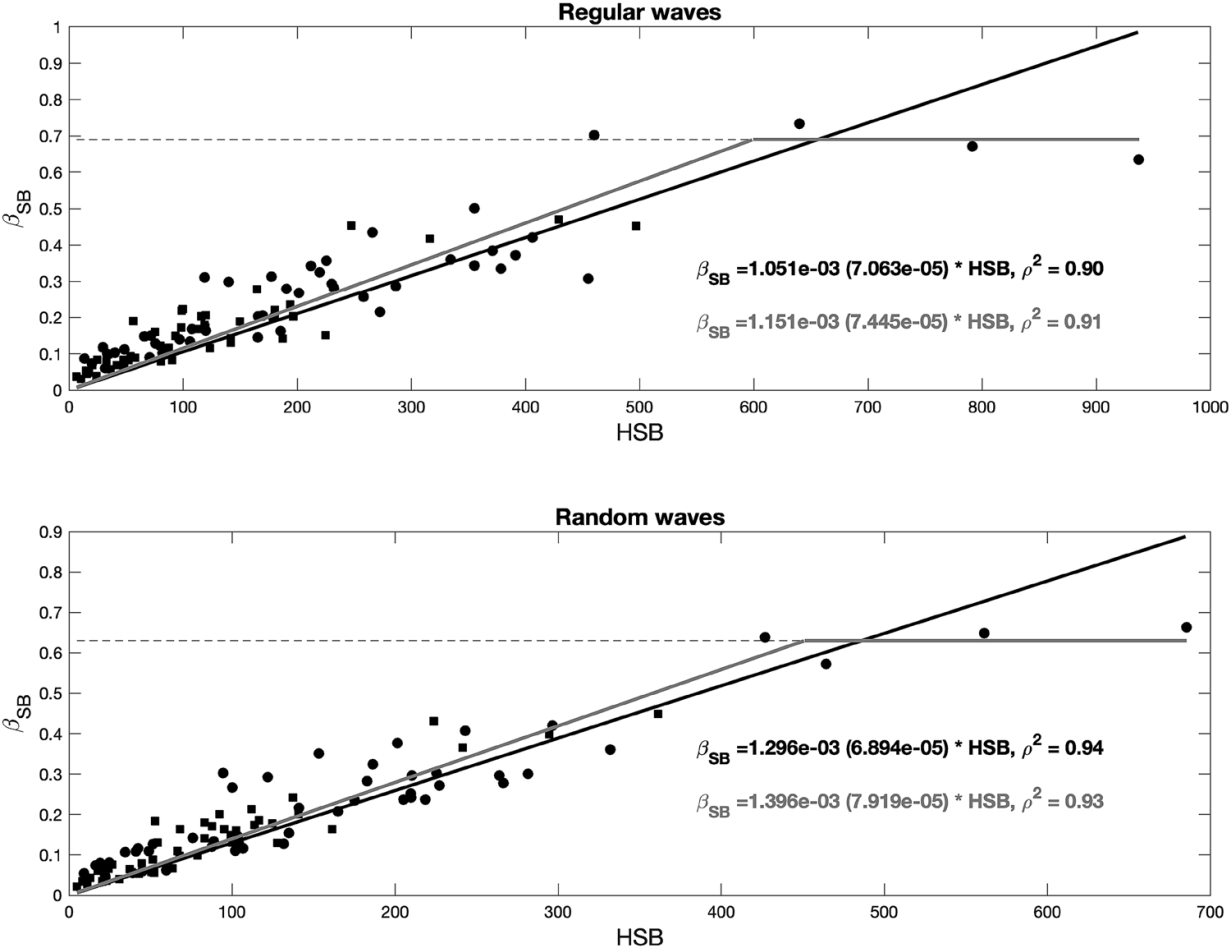
Wave damping coefficient, ${\beta }_{SB}$, as a function of the hydraulic standing biomass, $HSB$, under all regular (top panel) and random (bottom panel) wave conditions. Both panels show linear fitting results obtained without considering the saturation point, indicated by the black solid line, and those obtained considering the saturation point, indicated by the gray solid line. The black dashed line indicates the saturation point. 95% confidence interval is shown in brackets and correlation coefficient (${\rho }^{2}$) for each fit is also displayed.

All damping coefficients considered in the previous analysis were obtained without subtracting any additional source of dissipation such as bottom and wall friction. Previous works, such as^24^, highlighted the high importance of considering any other sources of wave dissipation besides the effect of vegetation elements when quantifying the wave height attenuation capacity. In this case, the flume walls were made of glass, and the friction induced by these walls could be considered negligible. However, bottom friction could be significant, as observed in tests run after removing all vegetation stems. Then, the wave damping coefficient obtained after subtracting the bottom friction contribution, ${\beta }_{SB}$, is studied here. Figure 7 shows the relationship obtained between this damping coefficient, ${\beta }_{SB}$, and hydraulic standing biomass, $HSB$.

A linear relationship was also obtained for ${\beta }_{SB}$, revealing correlation coefficients similar to those obtained when analyzing $\beta$. The obtained linear relationships under regular and random wave conditions are expressed as Eqs. (10) and (11), respectively, where values between brackets are the 95% confidence interval for each coefficient. A two-section fitting relationship, Eqs. (12) and (13), was also included considering the saturation regime obtained in the *Halimione* sp. 100% density and h = 0.20 m cases with a ${\beta }_{SB}=$ 0.69 and 0.63 under regular and random wave conditions, respectively.

10 $${\beta }_{SB}=1.051*{10}^{-3} \left(7.063\cdot {10}^{-5}\right)*HSB$$

11 $${\beta }_{SB}=1.296*{10}^{-3} \left(6.894\cdot {10}^{-5}\right)*HSB$$

12 $${\beta }_{SB}=\left\{\begin{array}{l}1.151\cdot {10}^{-3} \left(7.445\cdot {10}^{-5}\right)*HSB \\ 0.685 \;(0.047)\end{array}\right. \begin{array}{l} \;\; 0<HSB< 599\\ \;\; HSB>599\end{array}$$

13 $${\beta }_{SB}=\left\{\begin{array}{l}1.396\cdot {10}^{-3}\left(7.919\cdot {10}^{-5}\right)*HSB \\ 0.631 \;\left(0.055\right)\end{array}\right. \begin{array}{l}\;\; 0<HSB< 451\\ \;\;HSB>451\end{array}$$

As can be noted, the ${\beta }_{SB}$ values are significantly lower than those obtained for $\beta$, especially in the shallowest water depth cases where bottom friction is the highest, as discussed above. The estimation of $\beta$ and ${\beta }_{SB}$ allows two possible approaches to determine the wave damping effect of a saltmarsh. The first approach, based on $\beta$, includes wave damping induced by the combined effect of vegetation and bottom friction. Therefore, the consideration of $\beta$ in analytical or numerical analysis could provide the total dissipation induced by the species under study, and sediment characteristics are not necessary for analysis. Considering that saltmarsh species grow in muddy to sandy environments and that the major contribution to the obtained wave attenuation is associated with vegetation, this approach may be the best option if soil properties are not thoroughly characterized.

The second approach relies on the definition of ${\beta }_{SB}$. In this case, the wave damping contributions of vegetation drag and bottom friction are separated. Then, ${\beta }_{SB}$ can be used in cases where the effect of both momentum sinks can be separately evaluated. To quantify the wave damping contribution of vegetation drag only, ${\beta }_{SB}$ can be used, and then, the additional friction due to the bottom effect can be added considering the soil properties in each case. This second approach assumes a linear sum of both momentum sinks and could be applicable when soil properties are thoroughly characterized.

## Discussion

A total of 288 tests are presented and analyzed in this work, 144 tests under regular wave conditions and 144 tests under random wave conditions. The wave damping coefficient in each test is analyzed, and the damping coefficient accounting only for the effect of the standing biomass, ${\beta }_{SB}$, is determined. Consistent with previous works, higher wave attenuation rates are obtained at lower submergence ratios, shorter wave periods and larger wave heights. When analyzing the wave damping effect induced by bare soil, it can be observed that bottom friction-induced dissipation plays a more important role for the pioneer species, i.e., *Spartina* sp. and *Salicornia* sp., than for the upper marsh species, i.e., *Juncus* sp. and *Halimione* sp., which can dissipate wave energy to a greater extent.

The obtained damping coefficients are related to the measured standing biomass, leading to new relationships based on this vegetation variable. A new variable, the hydraulic standing biomass ($HSB$), is defined to consider the wide range of plant height values among species and incident wave conditions through the relative wave height and relative meadow length. Consideration of this variable results in a linear fit between $\beta$ and $HSB$ under both regular and random wave conditions. A saturation regime is reached for certain values of $HSB$, in which the wave height beyond the meadow can be assumed as negligible and the damping coefficient reaches an almost constant value. Then, a two-section fitting relationship accounting for this saturation regime is proposed, allowing us to estimate $\beta$ based on incident wave conditions and the meadow standing biomass and mean plant height. Additionally, the relationship between ${\beta }_{SB}$ and $HSB$ is analyzed to obtain fitting equations allowing estimation of wave damping produced by the isolated effect of the meadow standing biomass. A two-section fitting equation is also obtained by considering the saturation regime. It should be noted that the obtained relationships do not depend on any calibration coefficient and are solely a function of incident wave characteristics, standing biomass and mean plant height.

The established new damping coefficients, $\beta$ and ${\beta }_{SB}$, can be considered in analytical or numerical models as follows. The first damping coefficient, $\beta$, facilitates the determination of wave damping induced by the combined effect of vegetation and bottom friction. The consideration of $\beta$ in analytical or numerical models may be the best option if soil properties are not suitably characterized. The second damping coefficient, ${\beta }_{SB}$, can be used if wave damping induced by vegetation drag and bottom friction can be evaluated separately. The consideration of ${\beta }_{SB}$ instead of $\beta$ may be useful when soil properties are satisfactorily characterized, and wave damping induced by bottom friction can be accurately obtained.

This study presents a new approach to quantify wave damping due to saltmarshes, significantly reducing the number of variables needed to define meadows while avoiding the use of calibration coefficients, such as the drag coefficient widely used in existing approaches. Hence, wave damping can be directly determined if incident wave conditions and the meadow mean height and standing biomass are known. The last variable can be retrieved from aerial images or remote sensing data, which widely expands the applicability of this new approach. Additionally, this approach does not rely on any calibration coefficient and can be directly applied based on the abovementioned characteristics. This may represent a paradigm shift in the estimation of the wave energy attenuation effect of saltmarshes.

## Methods

### Vegetation species collection

*Spartina* sp. was collected in Santoña, *Salicornia* sp. in La Maruca, *Halimione* sp. in Oyambre and *Juncus* sp. in Tina Menor (panel A of Supplementary Fig. S1). Plants were collected and relocated into boxes of 0.19 × 0.29 m, including a 0.10 m sediment layer, to minimize the stress on the collected plants and to subsequently evaluate the flow energy damping effect of bare soil. Panel B of Supplementary Fig. S1 shows the different steps involved in vegetation collection with the sediment layer and placing samples in the boxes for transport. The process was performed by minimizing plant damage and maintaining the density found in the field.

### Meadow characteristics analysis

The standing biomass was measured across a total of 25 boxes (27% of the total number of boxes), namely, 5 boxes directly in the field, two samples of 5 boxes during the first cut and another two samples of 5 boxes during the second cut, to assess a representative subset^50–52^. The average dry weight of the standing biomass (g/m^2^) was measured after drying the plants for 48 h at 70 °C to a constant weight. This dry weight was chosen to determine the standing biomass based on^35^, where a relationship between this variable and wave attenuation was established.

The plant height exhibits high variability. Consequently, it is recommended to collect sufficient replicates to accurately characterize this plant trait^52^. In these experiments, 60 individual plants of each species were randomly selected to obtain an estimate of the plant height by directly measuring the height of each sample.

### Wave height attenuation analysis

The^18^ formulation, Eq. (14), was used for regular waves, and that of^19^, Eq. (2), was used for random waves:

14 $$\frac{H}{{H}_{i}}=\frac{1}{1+\beta X}$$

15 $$\frac{{H}_{rms}}{{H}_{rms,i}}=\frac{1}{1+\beta X}$$

where $H$ is the wave height for regular waves, ${H}_{rms}$ is the root mean square wave height for random waves, $\beta$ is the damping coefficient, $X$ is the position along the meadow with $X=0$ at the leading edge and subscript i denotes the incident value. β was fitted in each test based on the incident wave height recorded by free surface gauge 4, i.e., the gauge located directly offshore of the meadow (Fig. 1). The damping coefficient in the zero-density cases, ${\beta }_{B}$, was also obtained, and these values were used to obtain the damping coefficient isolating the effect of the standing biomass, ${\beta }_{SB}$, by relating the wave energy flux produced along the vegetation field to the dissipation induced by the meadow and bottom friction as follows:

16 $${\beta }_{SB}=\beta -{\beta }_{B}$$

where $\beta$ is the damping coefficient determined in the 100% and 50% density experiments, ${\beta }_{B}$ is the damping coefficient in the zero-density cases and ${\beta }_{SB}$ is the resultant damping coefficient accounting only for the effect of the standing biomass.

### Statement on plant materials

Experiments in this research were executed with four vegetation species (*Spartina maritima*, *Salicornia europaea*, *Halimione portulacoides* and *Juncus maritimus*) collected in Cantabrian estuaries with the permission of the Dirección General de Biodiversidad, Medio Ambiente y Cambio Climático of the Government of Cantabria. All plant experiments were carried out with accordance to relevant general regulations and guidelines and to the specific guidelines defined by the Dirección General de Biodiversidad, Medio Ambiente y Cambio Climático of the Government of Cantabria.

## Supplementary Information

Supplementary Information.

## Data Availability

The datasets generated during and/or analyzed during the current study are available from the corresponding author on reasonable request.
