# Supplementary material for: A paradigm shift in the quantification of wave energy attenuation due to saltmarshes based on their standing biomass
Source: Sci Rep. 2022 Aug 16;12:13883. doi: 10.1038/s41598-022-18143-6 (PMC9381759; doi:10.1038/s41598-022-18143-6)
Supplement: Supplementary file 1 — Supplementary Information. [file 41598_2022_18143_MOESM1_ESM.pdf]

Supplementary Information

A paradigm shift in the quantification of wave energy attenuation due to saltmarshes based on their standing biomass

Maria MAZA, Javier L. LARA, Iñigo J. LOSADA

Figure S1. Panel A (Map data: Google, Data SIO, NOAA, U.S. Navy, NGA, GEBCO, Google Earth Pro 7.3.4.8642, https://earth.google.com/web/) shows the four locations where vegetation was collected: 1. *Juncus sp.* in Tina Menor, 2. *Salicornia sp.* in La Maruca, 3. *Halimione sp.* in Oyambre and 4. *Spartina sp.* in Santoña. Panel B shows the different steps followed to collect the vegetation with the sediment layer and placing it in the boxes to be taken to the laboratory.

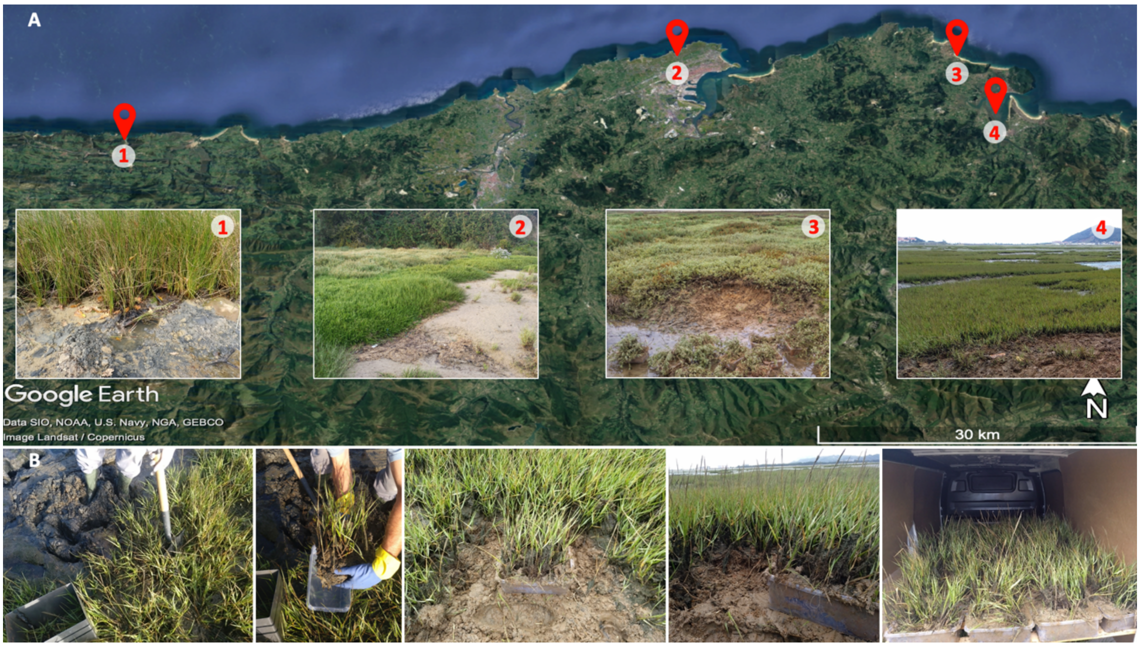

Figure S2. Three top pictures show 100, 50 and 0% density scenarios for *Spartina sp.* Bottom pictures display, from left to right, how the vegetation was cut (left picture) and the vegetation cut after the second cut for the four species: *Spartina sp., Salicornia sp., Juncus sp. and Halimione sp.*

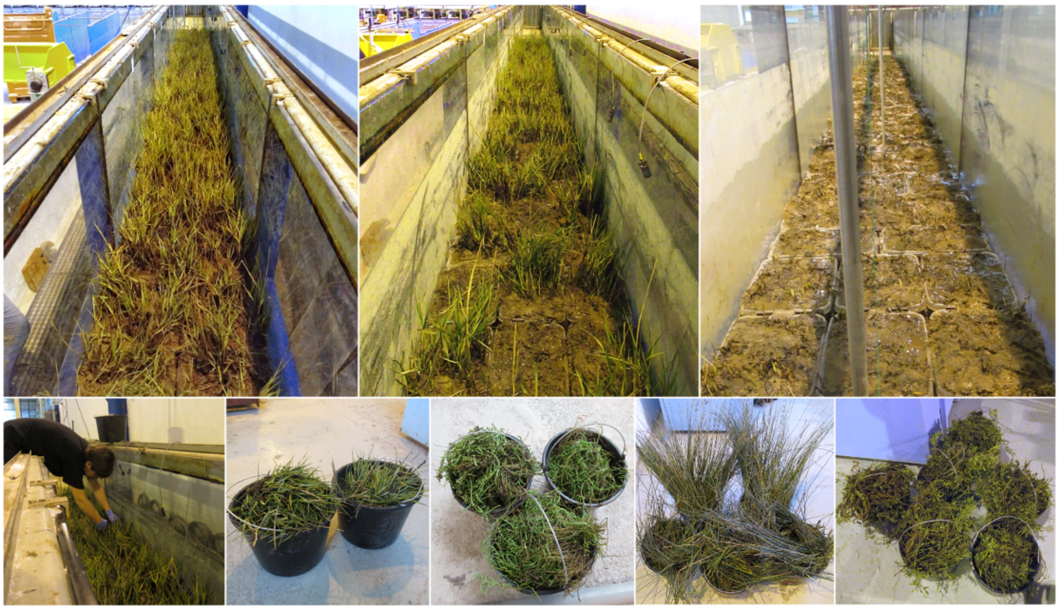

Figure S3. Wave damping analysis for the *Halimione sp.* 100% density case under regular, left column, and random, right column, wave conditions with h = 0.20 m.

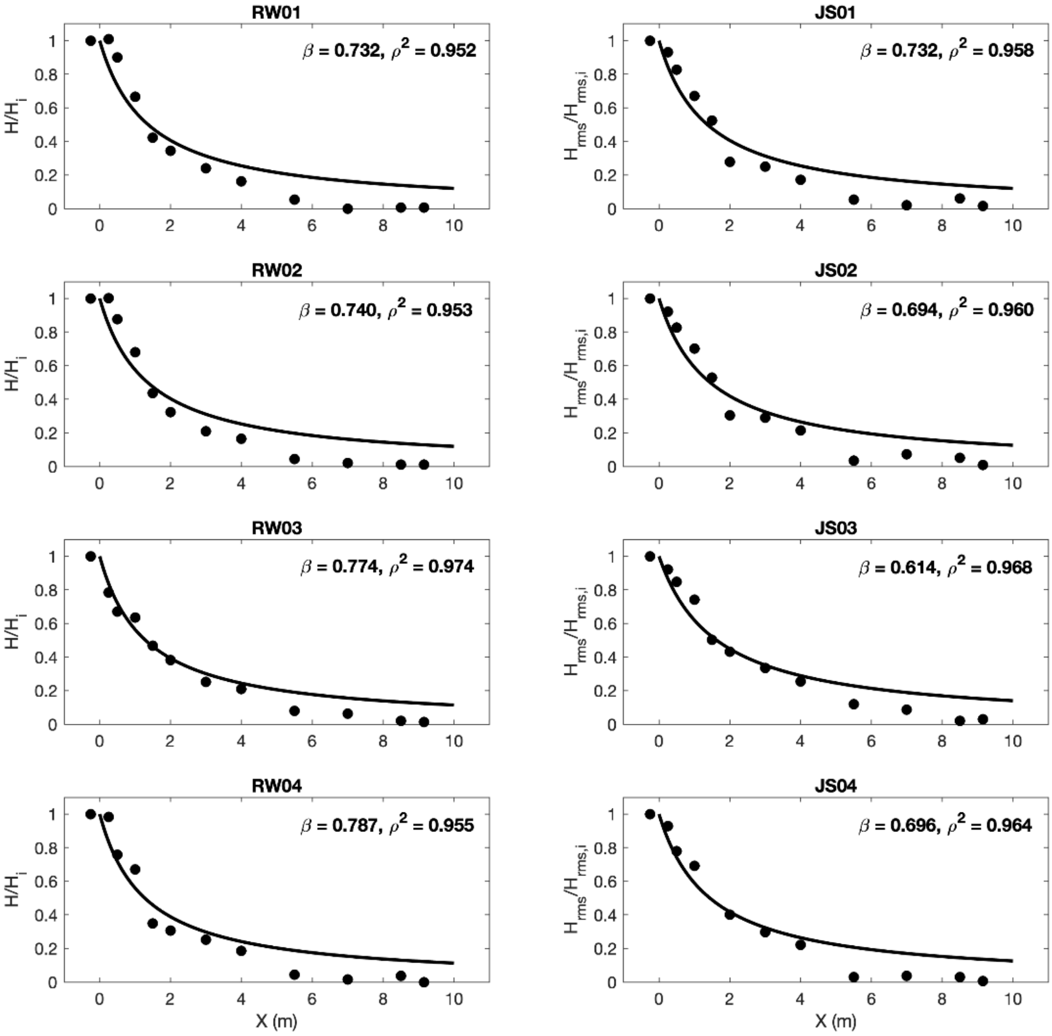

Table S1. Hydrodynamic conditions tested for the four vegetation species and the two densities per species. *RW* and *JS* denotes regular and random wave conditions, respectively. h is the water depth, H_i the incident wave height, T the wave period, $H_{S,i}$ the incident significant wave height and T_p the peak period.

Regular				*Random*			
Test	h (m)	H_i (m)	T (s)	*Test*	h (m)	$H_{S,i}$ (m)	T_p (s)
RW01	0.2	0.06	1.5	*JS01*	0.2	0.06	1.8
RW02	0.2	0.07	2.0	*JS02*	0.2	0.07	2.4
RW03	0.2	0.07	2.5	*JS03*	0.2	0.07	3.0
RW04	0.2	0.03	1.5	*JS04*	0.2	0.04	1.8
RW05	0.3	0.10	1.5	*JS05*	0.3	0.10	1.8
RW06	0.3	0.12	2.0	*JS06*	0.3	0.11	2.4
RW07	0.3	0.06	1.5	*JS07*	0.3	0.08	1.8
RW08	0.3	0.05	1.5	*JS08*	0.3	0.06	1.8
RW09	0.4	0.11	1.5	*JS09*	0.4	0.12	1.8
RW10	0.4	0.15	2.0	*JS10*	0.4	0.13	2.4
RW11	0.4	0.13	4.0	*JS11*	0.4	0.13	4.8
RW12	0.4	0.06	1.5	*JS12*	0.4	0.08	1.8

Table S2. Free surface gauges position with respect to the meadow leading edge (X = 0).

Gauge	*1*	*2*	*3*	*4*	*5*	*6*	*7*	*8*	*9*	*10*	*11*	*12*	*13*	*14*	*15*
X (m)	-4.50	-0.80	-0.60	-0.25	0.25	0.50	1.00	1.50	2.00	3.00	4.00	5.50	7.00	8.50	9.15

Table S3. Wave damping coefficients for tests performed with 100% density including the 95% confidence interval (95%CI) and the correlation coefficient ρ^2.

	Spartina sp.			*Salicornia sp.*			*Juncus sp.*			*Halimione sp.*		
Test	β	95%CI	ρ^2	β	95%CI	ρ^2	β	95%CI	ρ^2	β	95%CI	ρ^2
RW01	0.393	0.120	0.967	0.396	0.108	0.974	0.526	0.207	0.953	0.732	0.108	0.952
RW02	0.381	0.113	0.970	0.453	0.132	0.970	0.592	0.182	0.972	0.740	0.083	0.953
RW03	0.341	0.083	0.969	0.327	0.094	0.958	0.490	0.126	0.976	0.774	0.057	0.974
RW04	0.374	0.103	0.964	0.416	0.104	0.972	0.428	0.115	0.972	0.787	0.063	0.955
RW05	0.167	0.043	0.952	0.207	0.033	0.981	0.437	0.130	0.978	0.378	0.055	0.973
RW06	0.193	0.045	0.960	0.215	0.036	0.983	0.420	0.159	0.971	0.404	0.090	0.980
RW07	0.132	0.020	0.982	0.188	0.023	0.988	0.323	0.099	0.969	0.308	0.078	0.971
RW08	0.122	0.023	0.971	0.158	0.022	0.983	0.305	0.080	0.973	0.302	0.046	0.974
RW09	0.144	0.011	0.996	0.148	0.021	0.991	0.260	0.057	0.984	0.234	0.041	0.989
RW10	0.151	0.022	0.982	0.128	0.019	0.981	0.295	0.076	0.979	0.237	0.056	0.976
RW11	0.086	0.022	0.934	0.065	0.016	0.931	0.170	0.052	0.971	0.150	0.057	0.973
RW12	0.104	0.013	0.987	0.101	0.016	0.970	0.244	0.050	0.981	0.176	0.036	0.986
JS01	0.404	0.122	0.967	0.433	0.100	0.979	0.488	0.124	0.978	0.732	0.290	0.958
JS02	0.318	0.087	0.965	0.331	0.075	0.978	0.454	0.090	0.984	0.694	0.264	0.960

JS03	0.288	0.094	0.960	0.285	0.082	0.972	0.421	0.096	0.981	0.614	0.207	0.968
JS04	0.347	0.108	0.960	0.366	0.090	0.974	0.386	0.127	0.966	0.696	0.296	0.964
JS05	0.158	0.037	0.965	0.174	0.018	0.992	0.346	0.080	0.977	0.346	0.080	0.981
JS06	0.132	0.028	0.979	0.146	0.023	0.987	0.288	0.072	0.977	0.309	0.070	0.982
JS07	0.142	0.016	0.991	0.155	0.020	0.985	0.277	0.063	0.979	0.341	0.076	0.983
JS08	0.128	0.014	0.991	0.154	0.018	0.991	0.274	0.054	0.980	0.320	0.066	0.984
JS09	0.105	0.013	0.989	0.094	0.008	0.994	0.270	0.049	0.983	0.186	0.022	0.993
JS10	0.096	0.016	0.987	0.083	0.011	0.991	0.230	0.044	0.987	0.170	0.021	0.992
JS11	0.054	0.014	0.971	0.048	0.010	0.981	0.142	0.041	0.969	0.110	0.018	0.989
JS12	0.092	0.012	0.984	0.078	0.011	0.977	0.239	0.032	0.990	0.157	0.017	0.994

Table S4. Wave damping coefficients for tests performed with 50% density including the 95% confidence interval (95%CI) and the correlation coefficient ρ^2.

	Spartina sp.			*Salicornia sp.*			*Juncus sp.*			*Halimione sp.*		
Test	β	95%CI	ρ^2	β	95%CI	ρ^2	β	95%CI	ρ^2	β	95%CI	ρ^2
RW01	0.270	0.060	0.979	0.240	0.054	0.980	0.340	0.062	0.985	0.549	0.190	0.963
RW02	0.286	0.049	0.986	0.222	0.068	0.967	0.369	0.091	0.973	0.539	0.204	0.959
RW03	0.203	0.063	0.934	0.171	0.055	0.929	0.262	0.065	0.961	0.457	0.144	0.959
RW04	0.254	0.045	0.984	0.231	0.038	0.981	0.309	0.035	0.995	0.539	0.151	0.978
RW05	0.142	0.019	0.983	0.127	0.023	0.974	0.273	0.041	0.987	0.247	0.059	0.979
RW06	0.125	0.021	0.983	0.148	0.025	0.979	0.248	0.046	0.986	0.266	0.052	0.984
RW07	0.104	0.024	0.964	0.113	0.024	0.971	0.197	0.039	0.986	0.208	0.060	0.972
RW08	0.087	0.024	0.949	0.102	0.026	0.955	0.186	0.041	0.973	0.195	0.055	0.973
RW09	0.093	0.012	0.994	0.115	0.019	0.990	0.187	0.018	0.993	0.152	0.024	0.988
RW10	0.088	0.014	0.971	0.094	0.013	0.980	0.154	0.034	0.977	0.157	0.028	0.980
RW11	0.036	0.013	0.856	0.050	0.015	0.888	0.096	0.026	0.944	0.099	0.019	0.966
RW12	0.062	0.007	0.980	0.071	0.011	0.967	0.140	0.016	0.989	0.125	0.016	0.988
JS01	0.233	0.045	0.980	0.236	0.038	0.986	0.310	0.056	0.985	0.518	0.173	0.970
JS02	0.190	0.035	0.980	0.182	0.030	0.987	0.261	0.055	0.981	0.444	0.109	0.976
JS03	0.152	0.030	0.984	0.165	0.032	0.984	0.245	0.052	0.977	0.407	0.094	0.977
JS04	0.228	0.047	0.974	0.200	0.029	0.986	0.232	0.029	0.992	0.489	0.153	0.972
JS05	0.108	0.008	0.995	0.113	0.013	0.991	0.227	0.029	0.989	0.246	0.041	0.987
JS06	0.086	0.012	0.992	0.094	0.017	0.985	0.185	0.027	0.988	0.211	0.034	0.989
JS07	0.091	0.004	0.997	0.095	0.011	0.989	0.201	0.021	0.991	0.225	0.036	0.988
JS08	0.082	0.005	0.995	0.088	0.012	0.987	0.180	0.020	0.990	0.201	0.034	0.986
JS09	0.068	0.005	0.993	0.072	0.003	0.997	0.156	0.014	0.993	0.130	0.011	0.994
JS10	0.059	0.006	0.993	0.059	0.007	0.992	0.121	0.017	0.988	0.113	0.015	0.990
JS11	0.022	0.007	0.948	0.027	0.009	0.962	0.064	0.017	0.973	0.066	0.014	0.983
JS12	0.053	0.004	0.991	0.057	0.003	0.996	0.132	0.018	0.983	0.103	0.014	0.989

Table S5. Wave damping coefficients for tests performed with zero density including the 95% confidence interval (95%CI) and the correlation coefficient ρ^2.

	Spartina sp.			*Salicornia sp.*			*Juncus sp.*			*Halimione sp.*		
Test	β	95%CI	ρ^2	β	95%CI	ρ^2	β	95%CI	ρ^2	β	95%CI	ρ^2
RW01	0.068	0.015	0.939	0.089	0.010	0.986	0.105	0.009	0.991	0.097	0.013	0.980
RW02	0.068	0.015	0.959	0.081	0.023	0.957	0.092	0.021	0.964	0.069	0.020	0.948
RW03	0.043	0.023	0.648	0.041	0.026	0.671	0.055	0.023	0.709	0.041	0.015	0.862
RW04	0.064	0.019	0.892	0.059	0.011	0.948	0.086	0.022	0.949	0.086	0.020	0.942
RW05	0.039	0.011	0.885	0.045	0.012	0.926	0.053	0.012	0.929	0.044	0.010	0.920
RW06	0.045	0.010	0.913	0.070	0.016	0.923	0.059	0.004	0.996	0.061	0.012	0.956
RW07	0.021	0.011	0.715	0.025	0.017	0.847	0.030	0.011	0.892	0.028	0.011	0.901
RW08	0.019	0.011	0.656	0.019	0.017	0.741	0.038	0.013	0.889	0.024	0.011	0.861
RW09	0.045	0.015	0.845	0.046	0.014	0.874	0.045	0.014	0.897	0.036	0.010	0.891
RW10	0.034	0.010	0.849	0.038	0.009	0.906	0.038	0.005	0.975	0.035	0.004	0.981
RW11	0.000	0.000	0.514	0.003	0.001	0.530	0.003	0.001	0.629	0.000	0.000	0.501
RW12	0.031	0.011	0.799	0.032	0.009	0.865	0.040	0.010	0.912	0.043	0.014	0.848
JS01	0.054	0.010	0.969	0.073	0.005	0.994	0.068	0.008	0.988	0.069	0.005	0.994
JS02	0.026	0.007	0.952	0.054	0.003	0.996	0.047	0.007	0.989	0.045	0.005	0.989
JS03	0.022	0.009	0.927	0.043	0.010	0.976	0.045	0.010	0.964	0.042	0.009	0.970
JS04	0.044	0.008	0.967	0.070	0.007	0.987	0.062	0.005	0.992	0.058	0.005	0.991
JS05	0.032	0.005	0.960	0.046	0.003	0.991	0.050	0.003	0.993	0.046	0.004	0.991
JS06	0.024	0.004	0.975	0.036	0.006	0.986	0.036	0.005	0.988	0.037	0.005	0.983
JS07	0.025	0.006	0.906	0.039	0.004	0.987	0.040	0.002	0.995	0.039	0.003	0.992
JS08	0.021	0.006	0.869	0.034	0.003	0.982	0.040	0.003	0.992	0.037	0.004	0.983
JS09	0.025	0.004	0.962	0.033	0.004	0.984	0.033	0.003	0.984	0.032	0.003	0.985
JS10	0.017	0.003	0.971	0.023	0.003	0.987	0.023	0.004	0.952	0.024	0.002	0.988
JS11	0.000	0.001	0.532	0.003	0.002	0.611	0.001	0.001	0.509	0.001	0.001	0.261
JS12	0.019	0.003	0.957	0.023	0.002	0.982	0.023	0.003	0.983	0.024	0.002	0.980
